# Comparison of self-administered vaginal misoprostol versus placebo for cervical ripening prior to operative hysteroscopy using a sequential trial design*

**DOI:** 10.1111/j.1471-0528.2007.01628.x

**Published:** 2008-01-16

**Authors:** KS Oppegaard, B-I Nesheim, O Istre, E Qvigstad

**Affiliations:** a Department of Gynaecology, Helse Finnmark, Klinikk Hammerfest Hammerfest, Norway; b Department of Obstetrics, Women and Children's Division, Ullevål University Hospital Oslo, Norway; c Department of Gynaecology, Women and Children's Division, Ullevål University Hospital Oslo, Norway

**Keywords:** Cervical ripening, misoprostol, premenopausal, postmenopausal, sequential trial

## Abstract

**Objective:**

To compare the impact of 1000 micrograms of self-administered vaginal misoprostol versus self-administered vaginal placebo at home on preoperative cervical ripening in both premenopausal and postmenopausal women before operative hysteroscopy.

**Design:**

Two separate but identical parallel, randomised, double-blind, placebo-controlled sequential trials, one in premenopausal women and one in postmenopausal women. The boundaries for the sequential trials were calculated on the primary outcomes of a difference of cervical dilatation ≥1 mm, with the assumption of a type 1 error of 0.05 and a power of 0.95.

**Setting:**

Norwegian university teaching hospital.

**Sample:**

Eighty-six women referred to outpatient operative hysteroscopy.

**Methods:**

The women were randomised to either 1000 micrograms of self-administered vaginal misoprostol or self-administered vaginal placebo the evening before outpatient operative hysteroscopy.

**Main outcome measures:**

Preoperative cervical dilatation (primary outcome), number of women who achieve a preoperative cervical dilatation ≥5 mm, acceptability, complications and adverse effects (secondary outcomes).

**Results:**

In premenopausal women, the mean cervical dilatation was 6.4 mm (SD 2.4) in the misoprostol group and 4.8 mm (SD 2.0) in the placebo group, the mean difference in cervical dilatation being 1.6 mm (95% CI 0.5–2.7). Among the premenopausal women receiving misoprostol, 88% achieved a cervical dilatation of ≥5 mm compared with 65% in the placebo group. Twelve percent of the women who received misoprostol were difficult to dilate compared with 32% who received placebo. Dilatation was also quicker in the misoprostol group. Misoprostol had no effect on cervical ripening in postmenopausal women compared with placebo, and 43% of the women were difficult to dilate. The trials were terminated after analysis of 21 postmenopausal women and 65 premenopausal women after reaching a conclusion on the primary outcome with only 28% of the number of women needed in a fixed sample size trial. Three of 45 women who received misoprostol experienced severe lower abdominal pain, and there was an increased occurrence of light preoperative bleeding in the misoprostol group. Most women did not experience misoprostol-related adverse effects. The majority (83% of premenopausal and 76% of postmenopausal women) found self-administered vaginal misoprostol at home to be acceptable. There were two serious complications in the premenopausal misoprostol group: uterine perforation with subsequent peritonitis and heavy postoperative bleeding requiring blood transfusion, but these were not judged to be misoprostol related. Complications were otherwise comparatively minor and distributed equally between the two dosage groups.

**Conclusions:**

One thousand micrograms of self-administered vaginal misoprostol 12 hours prior to operative hysteroscopy has a significant cervical ripening effect compared with placebo in premenopausal but not in postmenopausal women. Self-administered vaginal misoprostol of 1000 micrograms at home the evening before operative hysteroscopy is safe and highly acceptable, although a small proportion of women experienced severe lower abdominal pain. There is a risk of lower abdominal pain and light preoperative bleeding with this regimen, which is very cheap and easy to use.

*Please cite this paper as:* Oppegaard K, Nesheim B, Istre O, Qvigstad E. Comparison of self-administered vaginal misoprostol versus placebo for cervical ripening prior to operative hysteroscopy using a sequential trial design. BJOG 2008;115:663–e9.

## Introduction

Over the past 20 years, minimally invasive operative techniques have been introduced for treating intrauterine pathology. Operative hysteroscopy or resectoscopy is the most common method for treating intrauterine pathology, such as myomas and endometrial polyps. Endometrial resection is a standard treatment of abnormal uterine bleeding if less invasive procedures fail.^1^ The diameters of resectoscopes (usually Charriere 24 or 26) necessitate dilatation of the cervical canal to 10 or 11 mm prior to insertion of the instrument. The complications encountered during dilatation, such as cervical tears, creation of false passages, and uterine perforation, are reported to be mainly related to the difficulty of cervical dilatation in nulliparous and postmenopausal women.^2^ An audit of women who have undergone operative hysteroscopy in our department supports this^3^ and reports a 7.8% complication rate related to hysteroscopic resection of endometrial polyps.

Prevention of cervical injury and uterine perforation during termination of pregnancy has been demonstrated by preoperative cervical ripening^4^,^5^ and may be achieved either mechanically, such as with osmotic dilators,^6^ or biochemically with prostaglandins.^7^ Solid evidence supports the efficacy of misoprostol for cervical ripening in pregnant women before first-trimester suction curettage abortion.^7^,^8^

In contrast, misoprostol for cervical ripening in nonpregnant women to prevent cervical injury during dilatation is not well established. We found 17 reported randomised controlled trials published in English that evaluated the efficacy of misoprostol on cervical ripening in nonpregnant women published before 1 September 2006, after searching medical literature databases including Pubmed^9^ and EMBASE Ovid.^10^ The search terms used included ‘misoprostol’, ‘cervical ripening/priming’‘hysteroscopy’, and ‘operative hysteroscopy’. References from identified publications were manually searched and cross-referenced to identify additional relevant articles. The studies have shown different cervical response and outcomes.^11^–^28^ Most of the studies have separately, but not systematically, compared the effect on different groups of women, such as nulliparous women and postmenopausal women. Eight of the studies included less than 50 women,^12^,^13^,^16^,^18^,^20^–^22^,^25^ four did not compare the effect of misoprostol with a placebo,^23^–^25^,^27^ and ten of the trials appear to be underpowered or lacking a sample size calculation as regards to evaluating primary outcome measures.^11^–^13^,^16^–^18^,^20^–^22^,^26^ It appears that none of the trials has been designed and conducted in accordance with the CONSORT statement.^28^ The dosages used in the studies have varied between 200 and 1000 micrograms of misoprostol given between 2 and 24 hours before hysteroscopy, via oral, sublingual, and vaginal routes. A review by Crane and Healy^29^ concludes that in premenopausal women, misoprostol appears to be promising as a cervical ripening agent prior to hysteroscopy, although further research is needed to identify the ideal dose, route, and timing. Further research in postmenopausal women or those receiving gonadotrophin-releasing hormone (GnRH) agonists (a group perceived as having an additional risk factor for complications)^2^ has been recommended to determine whether misoprostol is effective in cervical ripening in this population. Trials that have used higher dosages of misoprostol, via the vaginal route, using the longest time interval between administration of misoprostol and hysteroscopy have tended to show more favourable outcomes regarding cervical ripening.

Judging cervical width in millimetres preoperatively with dilators used in clinical practice is a normal method of assessing the effect of cervical ripening.^12^–^17^,^19^–^27^ Less commonly, preoperative ripening is assessed by measuring cumulative dilatation force using a cervical tonometer.^11^,^13^,^16^ Cervical resistance to dilatation or complications encountered during the procedure are also parameters that can indicate effectiveness of cervical ripening. The aim of our study was to investigate whether 1000 micrograms of self-administered vaginal misoprostol 12 hours before operative hysteroscopy results in effective preoperative cervical ripening compared with placebo.

## Methods

The study protocol was designed according to the recommendations in the CONSORT statement^28^ and was submitted to *BJOG* for review before recruitment of women. The study was a randomised, controlled, double-blind one-centre study at a central university gynaecological outpatient department. All women referred to outpatient operative hysteroscopy at Ullevål University Hospital between 1 August 2006 and 20 April 2007 were sent an invitation to be included in the study. Women are referred to our hospital for operative hysteroscopy from private practising gynaecologists, GPs or other hospitals. The common presenting complaints are abnormal/postmenopausal uterine bleeding, endometrial polyps, submucous myomas, and infertility. The invitation for study participation was sent together with dates for a preoperative outpatient consultation. The study invitation included detailed information regarding the study, as well as an informed consent form. The outpatient consultation took place a few days prior to the scheduled operation, where the women received additional information and were given the option to participate in the study. Women who had a medical indication for operative hysteroscopy and who had given informed consent were eligible for study recruitment. Exclusion criteria were as follows: women who were unable to communicate in Norwegian, women without an indication for hysteroscopy, women who were medically unfit for surgery and women with a known allergy to misoprostol.

**Figure 1:**
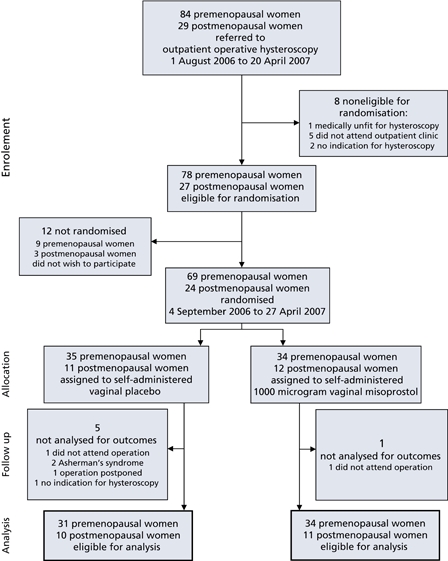
Flow diagram.

The study was carried out from 4 September 2006 until 27 April 2007 (Figure 1). Doctors examining women at the outpatient consultation (trainees and specialists) were responsible for recruiting the women. During the 8 months the study was carried out, 82% of the total number of premenopausal women and 79% of the total number of postmenopausal women referred to operative hysteroscopy participated in the study. The rest declined the offer (11% of the premenopausal and 10% of the postmenopausal women) or were excluded based on the exclusion criteria (7% of the premenopausal and postmenopausal women). Two separate, but identical, studies were conducted in parallel, based on the women's menopausal status. Each participant received either 1000 micrograms of misoprostol or placebo, which they self-inserted vaginally at least 12 hours before operative hysteroscopy. The randomisation was performed with permuted blocks, using the randomisation plan generator, as described by Dallal.^30^ The randomisation procedure was third party concealed randomisation, performed by the hospital pharmacist. Placebo misoprostol tablets are difficult to make; therefore, gelatine capsules with an identical appearance were manufactured by the hospital pharmacist. The active misoprostol was ground up as a whitish powder inserted into gelatine capsules (500 microgram misoprostol per capsule), as was an inactive ingredient, lactosum monohydricum—which has an identical appearance to ground misoprostol tablets—and subsequently inserted into gelatine capsules as placebo. The hospital pharmacist prepared numbered, opaque, sealed plastic containers labelled ‘Misoprostol 0.5 mg/Placebo, 2 vaginal capsules’. The prepared capsules were inserted into containers by the hospital pharmacist, which were sealed with tamper-proof seals. Each container contained two capsules. Half of the containers contained two capsules with 500 microgram misoprostol each, while the other half contained two placebo capsules. The containers were then delivered to the outpatient clinic. As the women were recruited, the doctor at the outpatient consultation recorded the preoperative variables on a standardised case report form (on page 1), and the women were given a plastic container containing the capsules before leaving the hospital. Hence, those involved in administering the intervention and the women were blinded to the treatment received. Each study participant opened a numbered container at home, containing either misoprostol or lactosum monohydricum in capsules. The women were instructed to insert the capsules as deep as possible vaginally after voiding urine at approximately 9 p.m. the evening before the operation.

On admission to the operating theatre, nurses recorded symptoms and comments from the women on the case report form (page 1). The women recorded pain experienced between the insertion of capsules and the operation on a visual analogue scale score, ranging from 0 (no pain) to 10 (unbearable pain). Women were asked to rate acceptability of self-administered capsules on a scale from 1 to 4 (1 = completely acceptable, 2 = fairly acceptable, 3 = fairly unacceptable, 4 = completely unacceptable). The women were then given a general intravenous anaesthetic (propofol/fentanyl/alfentanyl) by the attending anaesthesiology nurse, after which the nurses prepared the women for operation by disinfecting the vulval and vaginal area with a 0.05% chlorhexidine acetate solution (Fresenius Kabi, Halden, Norway). Visible vaginal capsule remains were noted before the area was irrigated. The case report form with the preoperative variables, recorded symptoms and comments from the women on page 1 was then turned over to page 2, so that this information was not available to the operating gynaecologist. The operators were then summoned to the operating theatre. They were thus both blinded to which treatment the women had received and to the occurrence of possible adverse effects from the treatment, as recorded by the nurses.

Six experienced senior gynaecologists (with 5–20 years experience in operative hysteroscopy) performed the operative hysteroscopies during the study period. Prior to operation of the first woman, the project leader individually instructed the operators in assessing preoperative cervical dilatation in order to obtain valid and reliable measurements. Before the operative hysteroscopy, the operator measured the preoperative degree of cervical dilatation by passing Hegar dilators through the cervix in ascending order starting with a size of 4 mm. The size of the largest dilator passed into the inner cervical ostium without subjective resistance felt by the operator was recorded as the preoperative degree of dilatation. If there was initial resistance with Hegar dilator of size 4 mm, then dilators of size 3 or 2 mm were tried. If there was resistance with Hegar dilator of size 2 mm, the result was recorded as 0 mm. After the cervical canal was dilated to a Hegar dilator of size 10 or 11 mm, an Olympus (Olympus, Hamburg, Germany) rigid resectoscope model A22026A (Charriere 26) equipped with a Hopkins 12° rigid fibre optic model A22001A was passed into the uterine cavity. A sodium chloride 9% solution (Baxter, Norfolk, UK) was infused for uterine irrigation. A bipolar diathermal current of 280 watts (pure cut) supplied by a Surgmaster® US-40 (Olympus) diathermia unit was routinely used for resection of pathological uterine masses (myomas, polyps, uterine septae, etc.) and endometrium. For haemostasis coagulation, a current of 80 watts was applied. Adverse events during the operation, such as superficial cervical lacerations, production of false passage of the cervix during cervical dilatation, and perforation of the uterus were recorded. The women's records were reviewed after 14 days for postoperative complications.

The null hypotheses for the trials were that there is no clinically significant difference in preoperative baseline cervical dilatation (<1 mm), between women who receive misoprostol and those who receive placebo. A sequential trial plan using a two-sample sequential Wilcoxon test developed by Skovlund and Walløe was used in the current study to keep the number of women needed to reach a conclusion as low as possible.^31^–^33^ This method has previously been used in clinical trials and has been shown to be easy to use.^34^,^35^ It is expected to reduce the number of women needed to reach a conclusion compared with the number required in a corresponding fixed sample trial. We performed a small pilot study in our department on 20 women prior to operative hysteroscopy in January 2006 to investigate the preoperative variability (SD) in cervical dilatation, in order to calculate the boundaries needed for the statistical model. The SD was 1.3 mm in postmenopausal women (*n* = 5) and 1.4 mm in premenopausal women (*n* = 15). The mean cervical dilatation was 3.4 mm in postmenopausal women and 5.4 mm in premenopausal women. The chosen boundaries for the statistical model were based on a significance level of 5% and a power of 95% in demonstrating a 1 mm difference in the cervical dilatation caused by misoprostol and placebo (the primary end-point). As it seemed unlikely that use of misoprostol could cause a constriction of the cervix, a one-sided test was chosen. When one of the boundaries was crossed, the trial was stopped. In this case, crossing the lower boundary would have meant that misoprostol was significantly superior to placebo and crossing the upper boundary would have meant that the two treatments had been equally effective. Secondary end-points were as follows: the number of women who achieve satisfactory cervical priming (cervical dilatation ≥5 mm); 5 mm was chosen as ‘satisfactory’, as this would permit insertion of a diagnostic hysteroscope without further dilatation. A preoperative cervical dilatation of 5 mm would also make it much easier to further dilate the cervix with Hegar dilators if necessary (for insertion of an operative resectoscope of 10–11 mm), decreasing the risk of creating a false passage, acceptability of self-administration of vaginal capsules at home, the number of dilatations judged as ‘easy’ or ‘difficult’ by the operator, and the frequency of complications, as registered by the nurses preoperatively and the operators intraoperatively. The trials were not designed nor powered to test the difference for the secondary outcomes. Following the sequential Wilcoxon test by Skovlund and Walløe, the estimator of normal means is applied if the observations are normally distributed.^36^

Approval from the Regional Committee for Medical Research Ethics in Northern Norway^37^ was granted for the study protocol on 23 May 2006. Permission from Ullevål University Hospital's Advisory Committee on the Protection of Patient Records was granted on 31 May 2006 and from the Norwegian Medicines Agency^38^ on 7 July 2006. The study protocol was published on http://www.clinicaltrials.gov on 17 August 2006 and was submitted to the European Clinical Trials Database during May 2006 and has previously been published in *BJOG*.^39^ Each participating woman was insured through the Drug Liability Association with liability insurance in connection with clinical trials of drugs.

## Results

**Figure 2:**
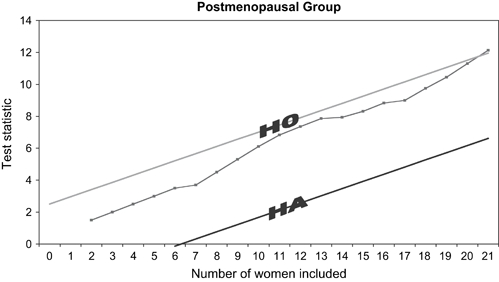
Continuation of the sequential test. The stopping boundaries and the sample path leading to the conclusion that misoprostol was not significantly different from placebo are shown. H0, boundary for the null hypothesis; HA, boundary for the alternative hypothesis.

**Figure 3:**
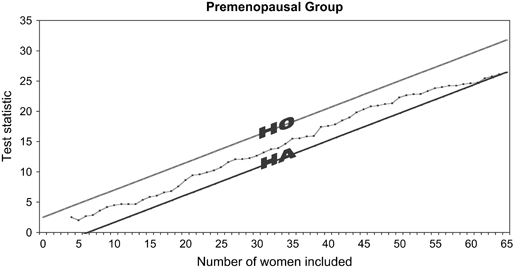
Continuation of the sequential test. The stopping boundaries and the sample path leading to the conclusion that misoprostol was significantly superior to placebo are shown. H0, boundary for the null hypothesis; HA, boundary for the alternative hypothesis.

The stopping boundaries were reached on 2 March (after 179 days) for the postmenopausal group (Figure 2), showing no significant difference between the placebo and misoprostol groups on the primary outcome of preoperative cervical dilatation, and on 20 April 2007 (after 235 days) for the premenopausal group (Figure 3), showing a significant difference between the misoprostol and placebo groups on the primary outcome. The two treatment groups were comparable regarding basal clinical preoperative characteristics (Table 1). The indications for operative hysteroscopy and the operative procedure in the two study groups are shown in Table 2. The cervical dilations in the two treatment groups are shown in Table 3. In the premenopausal women, the mean cervical dilatation was 6.4 mm (SD 2.4) in the misoprostol group and 4.8 mm (SD 2.0) in the placebo group, the mean difference in cervical dilatation being 1.6 mm (95% CI 0.5–2.7). The cervical dilatations were normally distributed in the premenopausal trial. In the postmenopausal women, the mean cervical dilatation was 4.9 mm (SD 1.5) in the placebo group and 3.4 mm (SD 2.7) in the misoprostol. The cervical dilatations were not normally distributed in the postmenopausal trial. The main adverse effect was lower abdominal pain (Table 4), but 51% of the women who received misoprostol experienced no pain, in contrast to 42% experiencing pain characterised as mild or moderate—less or equal to menstrual pain. Three women (7%) who received misoprostol reported severe lower abdominal pain but did not take analgesics or report on their symptoms prior to operation and study registration of complications.

**Table 1:** Demographic characteristics of women in the two study groups according to dosage

	Premenopausal women		Postmenopausal women	
Characteristic	Self-administered vaginal placebo (*n* = 35)	Self-administered vaginal misoprostol (*n* = 34)	Self-administered vaginal placebo (*n* = 11)	Self-administered vaginal misoprostol (*n* = 12)
Age (years), mean (SD)	42.11 (5.4)	43.38 (6.8)	62.64 (6.0)	63.3 (6.3)
Body mass index (kg/m^2^), mean (SD)	25.2 (4.8)	24.1 (4.3)	28.8 (6.2)	24.6 (5.3)
Women currently using hormone therapy, *n* (%)	3 (8.8)	5 (14.7)	3 (27.3)	5 (41.7)
Total number of children born, mean (SD)	1.3 (1.4)	1.6 (1.3)	1.6 (0.8)	1.4 (1.2)
Number of vaginal deliveries, mean (SD)	1.1 (1.3)	1.2 (1.3)	1.3 (0.9)	1.3 (1.1)
Women delivered with caesarean sections, *n* (%)	4 (11.4)	10 (29.4)	2 (18.2)	1 (8.3)
Women with previous cervical dilatation, *n* (%)	19 (54.3)	10 (29.4)	4 (36.4)	4 (33.3)
Women with previous cone biopsy, *n* (%)	3 (8.8)	3 (8.8)	1 (9.1)	1 (8.3)

**Table 2:** Indications for operative hysteroscopy and operative procedure in the two study groups according to dosage

	Premenopausal women		Postmenopausal women	
	Self-administered vaginal placebo	Self-administered vaginal misoprostol	Self-administered vaginal placebo	Self-administered vaginal misoprostol
**Referral reason**	**(*n* = 35), *n* (%)**	**(*n* = 34), *n* (%)**	**(*n* = 11), *n* (%)**	**(*n* = 12), *n* (%)**
Abnormal uterine bleeding	26 (74)	31 (91)	N/A	N/A
Postmenopausal bleeding	N/A	N/A	6 (55)	8 (67)
Asymptomatic endometrial polyp	4 (11)	0	3 (27)	4 (33)
Infertility	5 (14)	2 (6)	N/A	N/A
Asymptomatic submucous myoma	0	1 (3)	1 (9)	0
**Procedure**	**(*n* = 31), *n* (%)**	**(*n* = 34), *n* (%)**	**(*n* = 10), *n* (%)**	**(*n* = 11), *n* (%)**
Transcervical polyp resection	10 (32)	8 (24)	7 (70.0)	10 (91)
Transcervical myoma resection	8 (26)	12 (35)	2 (20.0)	0
Transcervical endometrium resection	12 (39)	12 (35)	0	0
Resection of uterine septum	1 (3)	1 (3)	N/A	N/A
No treatment	0	1 (3)	1 (10.0)	1 (9)

N/A, not applicable.

**Table 3:** Intraoperative findings and distribution of cervical dilatation in the two treatment groups

	Premenopausal women		Postmenopausal women	
Characteristic	Self-administered vaginal placebo (*n* = 31)	Self-administered vaginal misoprostol (*n* = 34)	Self-administered vaginal placebo (*n* = 10)	Self-administered vaginal misoprostol (*n* = 11)
**Mean difference in cervical dilatation (mm)**	1.6 (95% CI 0.5–2.7)		N/A	
**Cervical dilatation (mm)**				
Mean (SD)	4.8 (2.0)	6.4 (2.4)	4.9 (1.5)	3.4 (2.7)
Median (range)	5 (<2 to 8)	6 (<2 to 11)	5 (3 to 7)	4 (<2 to 7)
**Number of women achieving cervical dilatation ≥5 mm, *n* (%)**	20 (65)	30 (88)	7 (70)	3 (27)
**‘Difficult dilatation’, *n* (%)**	10 (32)	4 (12)	2 (20)	7 (64)
**Dilatation time (seconds), mean (SD)**	68 (59)	47 (24)	49 (29)	70 (48)
**Exposure to capsules (minutes), mean (SD)**	765 (92)	754 (101)	840 (103)	840 (118)
**Frequency of complications**	4	2	2	1

**Table 4:** Preoperative adverse effects in the two treatment groups and findings during treatment

	Premenopausal women		Postmenopausal women	
	Self-administered vaginal placebo (*n* = 31)	Self-administered vaginal misoprostol (*n* = 34)	Self-administered vaginal placebo (*n* = 10)	Self-administered vaginal misoprostol (*n* = 11)
Mean level of reported preoperative pain* (SD)	0.45 (1.2)	2.2 (2.5)	0.2 (0.6)	1.1 (2.8)
Occurrence of bleeding, *n* (%)	1 (3)	7 (21)	0	1 (8)
Shivering, *n* (%)	0	1 (3)	1 (10)	0
Diarrhoea, *n* (%)	0	1 (3)	0	0
Nausea, *n* (%)	1 (3)	0	0	1 (9)
Vaginal discharge, *n* (%)	1 (3)	0	0	0

* Measured with a visual analogue scale score, ranging from 0 (no pain) to 10 (unbearable pain).

Out of 67 premenopausal women, 46 (70%) found self-administered vaginal capsules at home to be completely acceptable, 9 (13%) fairly acceptable, 9 (13%) fairly unacceptable and 3 (4%) completely unacceptable. Out of 21 postmenopausal women, 11 (52%) found self-administered vaginal capsules at home to be completely acceptable, 5 (24%) fairly acceptable, 4 (19%) fairly unacceptable and 1 (5%) completely unacceptable. The main reason given for unacceptability was the lack of vaginal applicators aiding insertion.

There were a total of nine (11%) complications. Two serious complications occurred in women in the premenopausal misoprostol group who underwent transcervical myoma resections after the procedure was completed. One woman (who previously had one normal vaginal delivery and one caesarean section) sustained a uterine perforation that was not diagnosed during the operation. The woman was readmitted 4 days later with symptoms and signs of infection. A curettage raised suspicion of a perforation in the uterine fundus, which was then confirmed by subsequent laparoscopy. The other woman (who had previously had two vaginal deliveries) started to bleed heavily from the uterine wall after the myoma was removed during the operation. Haemostasis with coagulation did not stop the bleeding, so a balloon catheter was inserted and insufflated with 30 ml of saline for 12 hours. The bleeding subsequently subsided. Her haemoglobin level was 8.1 g/dl postoperatively, and she was offered and accepted blood transfusion with two red blood cell units. These complications were not judged to be misoprostol related. False passages through the cervix during cervical dilatation occurred in two women (one nulliparous and one with three vaginal deliveries) in the premenopausal placebo group. A false passage through the cervix and uterine perforation occurred in one woman in the postmenopausal misoprostol group, while a uterine perforation without further complications occurred in one woman in the postmenopausal placebo group. Cervical lacerations during the procedure occurred in one woman in the postmenopausal placebo group. Two women in the premenopausal placebo group were ambulatory treated with antibiotics for postoperative endometritis and a urinary tract infection, respectively. No further complications was reported.

## Discussion

Our trials show that 1000 microgram misoprostol self-administered vaginally by the woman 12 hours before operative hysteroscopy is safe and effective for cervical ripening compared with placebo in premenopausal but not in postmenopausal women. Self-administered vaginal capsules at home were considered highly acceptable; adverse effects were few and comparatively minor. We acknowledge that severe preoperative abdominal pain caused considerable anxiety and discomfort to the three women concerned. The study information form that each woman received prior to enrolment contained information on known possible adverse effects, including lower abdominal pain. However, we had not informed the women that they could use off-prescription analgesics if they experienced pain, and we revised our information to all subsequent trial participants. No women in the study used analgesics preoperatively.

The main strength of this study was that it was tailored to reach a conclusion on the primary outcome as soon as the difference was significant, so that as few women as possible were enrolled. A trial with a fixed sample size with the same primary end-point as ours would have required 151 premenopausal women (SD 2.4) and 151 postmenopausal women (SD 2.4) analysed in order to obtain a power of 95% based on a *t* test, assuming normally distributed observations and equal variance in the two groups. For ethical reasons, it is important to keep the number of women needed to reach a conclusion in clinical trials as low as possible. Our sequential trial reached a conclusion, after including only 28% of this patient number. In addition, premenopausal and postmenopausal women were analysed separately, and the conclusion was not given as part of a subgroup analysis. The study was designed and conducted strictly in adherence with the CONSORT criteria,^35^ and the protocol was submitted for peer review before the trial started including women. Furthermore, the study included all consecutive women referred to outpatient hysteroscopy and over 80% of women referred were included and analysed, giving a population-based study.

The main weakness was that six gynaecologists, not a single operator, were involved in assessing the primary outcome. Even though they were individually instructed in assessing preoperative cervical dilatation, it is difficult to be certain that every doctor's assessment was valid and reliable. The SD of the primary outcome probably increased with the number of operators. Furthermore, the sequential Wilcoxon test by Skovlund and Walløe is optimised for full randomisation, while we used block randomisation. These factors probably resulted in the trial needing more participants to reach a conclusion, resulting in a later curtailment. Two women were excluded from assessment and analysis after it was discovered preoperatively that they had uterine synechias (Asherman's syndrome) after they had been included. Furthermore, one woman was excluded from assessment and analysis because her intrauterine myoma was too large to be removed by resectoscopy. It was unfortunate that the women were not excluded before recruitment as not being eligible for hysteroscopy. However, the doctors recruiting the women at the outpatient clinic were not the same as the operating gynaecologists at the hysteroscopy. It was the operating gynaecologist's prerogative to decide whether the medical indication warranted hysteroscopy and whether the women should be analysed. However, the operators were blinded to which treatment the women had received, and we have no reason to suspect differential misclassification. The operations were planned independently of menstrual cycle and, consequently, while the trial was underway, we discovered an effect modifier that we had not considered prior to study commencement: some premenopausal women with bleeding disorders bled so profusely before insertion of the capsules, that the active ingredient most likely was ‘washed out’ of the vagina without having any effect on cervical ripening. These women were assessed on an intention-to-treat basis. Two of the three women who spontaneously remarked that the tablets most probably had been ‘washed out’ had a preoperative cervical dilatation of 2 mm or less.

Our audit in 2006^3^ supported previous studies that have identified women at risk of complications from operative hysteroscopy.^2^ GnRH agonist use, previous cone biopsy, and markedly retroverted uterus are additional risk factors for complications. It is not standard practice in Scandinavia to pre-treat with GnRH analogues and none of the women in our study had received them. In our current study, a substantial number of premenopausal women in the placebo group and postmenopausal women had a cervical dilatation that was judged as ‘difficult’ by experienced operators. We therefore believe that pre-treatment with this regimen in premenopausal women has the potential to facilitate dilatation, shorten the operation, and lower the risk of complications. Any randomised controlled trial designed to investigate complications would need a very large sample size and would probably be considered unethical. Misoprostol is a cheap drug and the cost of pre-treating prior to hysteroscopy would be negligible. Self-administration is easy and does not require hospital resources, other than information.

Misoprostol had no effect on cervical dilatation in postmenopausal women. A large number of the postmenopausal women referred to our outpatient clinic during this study were referred by their gynaecologists after failure to obtain an endometrial sample due to cervical stenosis. We speculate that whether the lack of estrogen is the main reason why misoprostol does not have any significant effect. We therefore feel that further investigations, as to whether a short course of local hormone therapy combined with misoprostol might have a positive cervical ripening effect on postmenopausal women, are warranted.

## Conclusions

One thousand micrograms of self-administered vaginal misoprostol administered 12 hours prior to operative hysteroscopy has a significant cervical ripening effect compared with placebo in premenopausal but not in postmenopausal women. We would recommend offering this inexpensive and easy to use regimen to nulliparous premenopausal women prior to undergoing operative hysteroscopy to reduce the risk of complications and facilitate cervical dilatation. Self-administered vaginal misoprostol of 1000 micrograms at home the evening before operative hysteroscopy is safe and highly acceptable, although a small proportion of women experienced severe lower abdominal pain. There is a risk of lower abdominal pain and light preoperative bleeding with this regimen and women should be made aware of this and offered standard analgesics.

## Funding

There was no pharmaceutical company involved in this study. The trial was funded by the normal running costs of the Department of Gynaecology at Ullevål University Hospital. In addition, a research grant of NOK 392 899 from the Regional Research Board of Northern Norway was awarded to finance leave of Dr K.S.O. from Hammerfest hospital as well as travel expenses between Hammerfest and Oslo and research courses. The research grant of Prof E.Q. (University of Oslo) funded the manufacturing costs of misoprostol and placebo capsules from the hospital pharmacy, as well as the costs incurred for preparing the randomisation schedule and distribution of containers containing capsules to hospital. The research grant of Prof E.Q. also funded insurance cover for the study participants.

The funding sources had no input into the study design, collection, analysis or interpretation of the data, report preparation or in the decision to submit the paper for publication. The corresponding author as well as B.-I.N. had full access to all the data in the study and had final responsibility to submit for publication.
